# Effects of exercise interventions on inflammatory biomarker levels in older adults with frailty and/or sarcopenia: a systematic review and meta-analysis

**DOI:** 10.3389/fimmu.2026.1734359

**Published:** 2026-04-30

**Authors:** Rui Chu, Yeshou Xie, Wenchao Li, Yinuo Du, Tao Ni, Xinyu Tu, Baoru Xu, Junqing Zhao

**Affiliations:** 1 Anhui Polytechnic University, Wuhu, Anhui, China; 2 Capital University of Physical Education and Sports, Beijing, China; 3 Changzhou University, Changzhou, Jiangsu, China

**Keywords:** exercise, frailty, inflammatory biomarker, meta-analysis, sarcopenia

## Abstract

**Objective:**

Excessive secretion of inflammatory biomarkers exerts adverse effects on muscle strength and function, and is associated with the development and progression of sarcopenia and frailty. For the improvement of inflammatory biomarker concentrations, given the remaining limitations of current pharmacological and nutritional therapies, exercise intervention may serve as a potential non-pharmacological intervention. The purpose of this study is to systematically evaluate the effects of exercise interventions on serum inflammatory biomarker levels in older adults with frailty and/or sarcopenia by employing a meta-analysis approach.

**Methods:**

A systematic search was conducted in PubMed, Web of Science, Cochrane Library, EMBASE, and Scopus (from database inception to October 2025) to identify randomized controlled trials (RCTs) investigating the impact of exercise interventions on serum inflammatory biomarker levels in older adults with frailty and/or sarcopenia. Data were analyzed using RevMan 5.4 and Stata 15.1. A random-effects model was employed to calculate standardized mean differences (SMD) and 95% confidence intervals (95% CI). The study protocol was registered with PROSPERO (CRD420251160555).

**Results:**

A total of 21 studies (comprising 26 RCTs) were included, involving 1,297 participants (intervention group: 659; control group: 613) with an age range of 63.6 to 86.7 years.Meta-analysis results revealed that exercise interventions did not significantly improve serum interleukin-6 (IL-6) (SMD = -0.04, 95% CI: -0.17 to 0.09, p = 0.52, I² = 10%) or C-reactive protein (CRP) concentrations (SMD = -0.08, 95% CI: -0.25 to 0.08, p = 0.32, I² = 0%) in older adults with sarcopenia and/or frailty. However, exercise interventions exerted a significant improving effect on tumor necrosis factor-α (TNF-α) (SMD = -0.31, 95% CI: -0.44 to -0.18, p < 0.0001, I² = 37%).

**Conclusion:**

Exercise interventions significantly improve TNF-α concentrations but do not exert a significant improving effect on IL-6 or CRP in older adults with frailty and/or sarcopenia. These findings may provide key evidence-based support for clinical non-pharmacological interventions.

**Systematic Review Registration:**

https://www.crd.york.ac.uk/PROSPERO/view/CRD420251160555, identifier CRD420251160555.

## 1 Introduction

Sarcopenia is a geriatric syndrome defined by progressive, generalized loss of muscle mass and strength, accompanied by impaired balance, which elevates the risk of falls, physical disability, mortality, and other adverse health outcomes (1). This condition exhibits clinical heterogeneity and frequently coexists with other conditions (e.g., osteoporosis, obesity), leading to combined phenotypes namely osteosarcopenia, sarcopenic obesity, and osteosarcopenic obesity (2–4).Epidemiological data indicate that the prevalence of sarcopenia among adults aged ≥ 60 years ranges from 10% to 27% (5), and exhibits a significant upward trend with increasing age. Among adults aged ≥ 80 years, the prevalence further exceeds 50% (6).Frailty, on the other hand is characterized as a state of reduced physiological reserve, blunted stress responsiveness, and disrupted homeostasis in older adults, typically manifesting with multisystem impairments such as decreased mobility, unintended weight loss, and cognitive decline (7, 8). Sarcopenia is widely regarded as an early phenotypic manifestation of frailty, sharing key pathological drivers including chronic low-grade inflammation, hormonal dysregulation, malnutrition, and sedentary lifestyles (9). Clinically, both conditions are marked by functional deterioration—most notably impairments in balance, muscle function, and muscle strength (9).Amid global population aging, the prevalence of sarcopenia and frailty is steadily increasing (10–13), and both are strongly linked to adverse health outcomes such as falls, hospitalization, progression of chronic diseases, and all-cause mortality (14–16). Given their substantial impact on geriatric health and healthcare systems, the development of effective strategies for preventing and managing frailty and sarcopenia has emerged as a critical clinical priority to enhance healthy aging and optimize healthcare resource utilization.

Inflammatory biomarker are a class of soluble signaling proteins primarily produced by immune cells and certain non-immune cells (17, 18). Their core physiological function is to initiate, amplify, and regulate inflammatory responses (19). Among these, tumor necrosis factor-α (TNF-α) is mainly produced by activated macrophages and monocytes, and serves as a key initiator in the early stages of the inflammatory cascade (20). Its signaling primarily occurs via binding to two cell surface receptors (TNFR1 and TNFR2); the formation of TNF-α-receptor complexes activates multiple downstream signaling pathways, with the nuclear factor kappa B (NF-κB) pathway being the most critical (21, 22). Activation of the NF-κB pathway enables its translocation into the nucleus, where it initiates the transcription of a series of proinflammatory genes—including other cytokines (e.g., interleukin-6 [IL-6]), chemokines, and adhesion molecules—thereby coordinating and amplifying the inflammatory response (23). Interleukin-6 (IL-6) is a pleiotropic cytokine produced by various cell types (e.g., neutrophils, macrophages, T cells, and even muscle cells) under stress conditions (24). IL-6 can bind to the membrane-bound IL-6 receptor (mIL-6R), which is expressed on a limited subset of cells (e.g., hepatocytes, some immune cells). The resulting complex then binds to the widely expressed signal transducer glycoprotein 130 (gp130), activating the downstream Janus kinase/signal transducer and activator of transcription (JAK/STAT) pathway. This pathway is primarily associated with tissue regeneration, metabolic regulation, and anti-inflammatory effects in the body (25, 26).C-reactive protein (CRP) is a classic acute-phase reactant protein synthesized exclusively in the liver (27). Its prominent role as an inflammatory biomarker stems from its stability and reliability as a systemic inflammation “indicator”: it directly reflects the activity of upstream proinflammatory factors (e.g., IL-6), making it one of the gold standards for clinically assessing systemic inflammatory burden (28).Within the complex inflammatory regulatory network, TNF-α, IL-6, and CRP play distinct yet interconnected hierarchical roles. TNF-α is typically regarded as one of the “first-wave signals” of the inflammatory response, and together with molecules such as interleukin-1β (IL-1β), constitutes the initiating components of the inflammatory cascade (29). IL-6 occupies a central hub position in the inflammatory network: it acts not only as a downstream effector molecule of upstream signals (e.g., TNF-α) but also as a key bridge linking innate immunity to adaptive immunity, and local inflammation to systemic responses (30). CRP lies at the end of this signaling axis, serving as a terminal product and stable biomarker of the inflammatory response (31). Collectively, these three molecules form a classic “TNF-α → IL-6 → CRP” signaling axis, which clearly illustrates the hierarchical progressive process of inflammation—from local initiation to systemic response (32).

Aging is not a passive degenerative process but is accompanied by a series of active physiological changes, one of which is inflammaging (33). This concept describes a non-infectious, chronic, low-grade systemic inflammatory state that emerges with advancing age. Its core biological feature is the sustained elevation of proinflammatory cytokines (e.g., IL-6, TNF-α) and CRP levels in the bloodstream, coupled with a relative reduction in anti-inflammatory cytokine levels (34). Elevated levels of CRP, TNF-α, and IL-6 can activate multiple signaling pathways, promote the infiltration of inflammatory cells, reduce protein anabolism, and increase protein catabolism—ultimately contributing to the development of sarcopenia and frailty (21). Additionally, increased inflammatory cytokine levels decrease the concentrations of growth factors and insulin-like growth factor-1 (IGF-1), further inhibiting protein synthesis (35). Inflammatory cytokines also induce myocyte apoptosis and autophagy, which exacerbate muscle mass loss, myocyte damage, and dysfunction (36). Meanwhile, the processes of muscle atrophy and fibrosis generate damage-associated molecular patterns (DAMPs). These signals recruit and activate immune cells (particularly macrophages) to infiltrate muscle tissue (37). The activated macrophages then switch to a proinflammatory phenotype and secrete large amounts of TNF-α and IL-6, transforming the muscle tissue itself into an active “inflammatory organ” and further facilitating the spread of inflammation from local to systemic levels (37, 38). Furthermore, numerous clinical studies have demonstrated that inflammatory cytokine levels exhibit a negative correlation with muscle mass and strength (39–41). Elevated serum IL-6 and TNF-α levels not only predict the onset of sarcopenia but also forecast adverse clinical outcomes of sarcopenia and frailty, such as disability and mortality (42).

While pharmacological interventions [e.g., non-steroidal anti-inflammatory drugs (NSAIDs), somatostatin analogs] and nutritional strategies [e.g., omega-3 fatty acids, vitamin D, vitamin C, and other antioxidants] can alleviate inflammatory responses to a certain extent, they are limited by poor adherence, high costs, and potential side effects (43–45). Compared with pharmacological and nutritional interventions, exercise training is a safer and more cost-effective alternative. Studies have shown that resistance training (e.g., weightlifting, squats, push-ups) helps improve systemic blood circulation, enhance metabolic function, induce muscles to secrete anti-inflammatory cytokines, and inhibit protein catabolism—thereby alleviating inflammatory responses (46). A meta-analysis further demonstrated that resistance training can effectively reduce serum IL-6 and CRP levels in healthy older adults (47); additionally, another study found that older adults who completed 12 months of moderate-intensity aerobic training exhibited reduced serum inflammatory cytokine levels compared to those who received only health education (48).

However, whether exercise interventions can effectively alleviate inflammatory responses and reduce serum CRP, IL-6 and TNF-α levels in older adults with frailty and/or sarcopenia remains a matter of some controversy. One study found that 61 frail older adults exhibited no significant changes in serum IL-6 and TNF-α levels following 24 weeks of exercise intervention (49); another study similarly reported that 25 individuals with sarcopenia showed no significant changes in serum CRP levels after 24 weeks of exercise intervention (50). Conversely, other studies have yielded positive results: one study demonstrated that individuals with sarcopenia exhibited significant reductions in serum IL-6 and TNF-α concentrations after 12 weeks of resistance training, regardless of whether the training was combined with nutritional intervention (51); another study found that 17 female individuals with sarcopenia experienced significant decreases in serum CRP concentrations following 8 weeks of kettlebell training (52). These significant heterogeneities in intervention effects across studies, coupled with inherent methodological limitations of individual RCTs—such as small sample sizes and high variability in intervention protocols—indicate that large-sample meta-analyses, which synthesize results from multiple studies, are urgently needed at this stage. This approach will more intuitively and clearly verify the effect of exercise interventions on serum inflammatory cytokine levels in older adults with frailty and/or sarcopenia.

Based on the above analysis, to address the existing research gaps to a certain extent, this study employs meta-analysis to quantitatively synthesize evidence from existing RCTs, with the goal of evaluating the intervention effects of exercise training on serum inflammatory markers in older adults with frailty and/or sarcopenia. Furthermore, a systematic review is conducted to explore the underlying mechanisms by which exercise modulates serum inflammatory marker levels. Additionally, subgroup analyses are performed to clarify two key aspects: first, differences in the intervention effects of three distinct exercise modalities on inflammatory markers, and second, variations in effectiveness between individuals with sarcopenia versus those with frailty. By integrating the aforementioned methods, this study aims to address the limitations of existing RCTs—such as small sample sizes and high variability in intervention parameters—and provide evidence-based support and practical guidance for the prevention of these conditions and the design of future clinical trials.

## 2 Methods

This study was conducted in accordance with the PRISMA (Preferred Reporting Items for Systematic Reviews and Meta-Analyses) statement, which provides standard guidelines for conducting systematic reviews and meta-analyses (53). The protocol has been registered in the PROSPERO database of the Centre for Reviews and Dissemination at the University of York, with the registration number.

### 2.1 Search strategy

The articles search process involved independent screening by multiple reviewers (YSX and YND). The retrieval time span covers from the establishment of each database to Oct 3, 2025, and the retrieval scope includes electronic databases such as Web of Science, Cochrane Library, PubMed, EMBASE, Scopus, and other relevant electronic databases. The search strategy adopted a combination of subject terms and free terms, covering the following concept groups: 1) Terms related to inflammatory markers; 2) Terms related to exercise intervention; 3) Terms related to the target population (older adults + frailty/sarcopenia). A detailed list of specific search terms and Search Strategy is provided in **Tables 1** and **2**.

**Table 1:** List of specific search terms.

Concept groups	Search terms
inflammatory markers	Somatomedin C, Cytokine, Chemotactic, Chemotactic Cytokines, Interleukin-6, Interleukin 6, B Cell Stimulatory Factor-2, IL-6, Tumor Necrosis Factor-alpha, Tumor Necrosis Factor alpha, TNF-alpha, C-Reactive Protein, C Reactive Protein, hs-CRP, hsCRP, Interleukin-10, Interleukin 10, IL-10, IL10, TGF-beta, TGFbeta, Interleukin 1 Receptor Antagonist Protein, IL-1 Inhibitor, IL-1Ra
Exercise Intervention	Exercise, Physical Exercise, Aerobic Exercise, Blood Flow Restriction Therapy, High-Intensity Interval Training, Resistance Training, Endurance Training
Target population	senior citizen, Aged, Elderly, Frailty, Sarcopenia, Frailties, Frailness, Frailty Syndrome, Sarcopenias
Research type	Randomized controlled trial

**Table 2:** List of search syntax.

Search strategy	Search terms
#1	Pro-Inflammatory/OR Cytokines/OR pro inflammatory cytokine/OR somatomedin c/OR chemotactic cytokine/OR cytokine chemotactic/OR Intercrine/OR chemotactic cytokines/OR Cytokines/OR Chemotactic/OR Intercrines/OR Chemokine/OR Interleukin-6/OR Interleukin-6/OR IL-6/OR IL6/OR tumor necrosis factor alpha/OR TNF-alpha OR tumor necrosis factor/OR c reactive protein/OR c reactive protein/OR hs-CRP/OR hsCRP/OR high sensitivity c reactive protein/OR Interleukin-10/OR Interleukin-10/OR IL-10/OR platelet transforming growth factor/OR bone derived transforming growth factor/OR TGF-beta/OR TGFbeta/OR interleukin 1 receptor antagonist protein/OR il 1 inhibitor/OR Urine/OR urine il 1 inhibitor/
#2	Exercise/OR Physical Exercise/OR Aerobic Exercise/OR Blood Flow Restriction Therapy/OR High-Intensity Interval Training/OR Resistance Training/OR Endurance Training/
#3	senior citizen/OR Aged/OR Elderly/
#4	Frailty/OR Frailties/OR Frailness/OR Frailty Syndrome
#5	Sarcopenia/OR Sarcopenias/
#6	Randomized controlled trial
#7	#4 OR #5
#8	#1 AND #2 AND #3 AND #6 AND #7

After literature retrieval, duplicate articles were first removed using EndNote (a reference management software). Then, titles and abstracts were initially screened based on pre-defined inclusion and exclusion criteria. Obviously irrelevant studies were excluded. Full texts were obtained for articles that passed the initial screening. Two independent researchers (YSX and YND) separately performed secondary screening, quality assessment, and data extraction. In case of disagreements, the corresponding author organized a discussion meeting to resolve them through negotiation.

### 2.2 Inclusion and exclusion criteria

Based on the PICOS principle of Cochrane, the inclusion and exclusion criteria for articles formulated in this study are as follows:

**Inclusion criteria:**

1. Study participants

Study participants included adults aged ≥60 years (regardless of gender) from all settings (e.g., communities, nursing homes, hospitals) who met the clinical diagnostic criteria for sarcopenia and/or frailty.

2.Sarcopenia diagnostic criteria:

Two sets of criteria were applied:

(1) EWGSOP2 (2019): Grip strength < 27 kg (males) or < 16 kg (females); appendicular lean mass (ALM) via dual-energy X-ray absorptiometry (DXA) < 7.0 kg/m² (males) or < 5.5 kg/m² (females); ALM via bioelectrical impedance analysis (BIA) < 7.0 kg/m² (males) or < 5.7 kg/m² (females); and gait speed ≤ 0.8 m/s (54);

(2) AWGS (2019): Grip strength < 28 kg (males) or < 18 kg (females); gait speed < 1.0 m/s; ALM via DXA < 7.0 kg/m² (males) or < 5.4 kg/m² (females) (55).

3. Frailty diagnostic criteria:

Three tools were used for assessment:

1. Frailty Phenotype (2001): Five criteria were evaluated (unintentional weight loss, self-reported fatigue, reduced grip strength, slow gait speed, inadequate physical activity). Participants were classified as frail (≥3 criteria met), pre-frail (1–2 criteria met), or robust (0 criteria met) (56);
2. Frailty Index (FI): Calculated as the proportion of ≥30 health deficits (e.g., symptoms, comorbidities, functional impairments). Frailty was defined as FI ≥ 0.25 (57);
3. Clinical Frailty Scale (CFS, 2005): Frailty severity was quantitatively assessed via scoring across domains (self-perceived capacity, ambulatory ability, cognitive function, emotional status). The CFS comprises 9 grades (ranging from “very fit” to “terminally ill”); grades 1–3 = robust, grades 4–5 = pre-frail, grades ≥6 = frail (58).

4. Experimental group interventions

The experimental group received interventions involving single or multiple exercise modalities, including:

Aerobic training (e.g., brisk walking, stationary cycling, running, calisthenics);
Resistance training (e.g., resistance exercises using various equipment);
Combined training (simultaneous implementation of aerobic and resistance training in a single session).
Only studies investigating the chronic effects (intervention duration≥4 weeks) were included.

5. Control group interventions

Participants in the control group received interventions including health education, routine care, and usual physical activity (i.e., maintenance of baseline activities of daily living). These interventions differed from those in the experimental group in terms of intervention type or target.

6. Study design

Only randomized controlled trials (RCTs) were included.

7. Outcome measures

Outcome measures involved in the study shall include at least one of IL-6, CRP, and TNF-α.

8. Language restriction

No language restrictions were applied to included studies to ensure comprehensive capture of all relevant literature.

Exclusion criteria:

1. Non-randomized controlled trials (non-RCTs).
2. The experimental group’s intervention measures were inconsistent (e.g., incorporating additional pharmaceutical interventions, or lacking clear definition of exercise type).
3. Study participants did not meet the criteria, including: non-human subjects; average age of participants was < 60 years; or no reporting of diagnostic criteria/guidelines for frailty and sarcopenia.
4. Study data were incomplete and irreplaceable (i.e., unable to be supplemented).
5. The relevant outcome indicators were not reported in the study results, or the corresponding data were non-extractable.
6. Grey literature, including unpublished manuscripts, conference proceedings, dissertations, theses, and other similar materials.

### 2.3 Data extraction

Two reviewers (YSX and YND) independently performed data extraction. If disagreements arose during the process, the corresponding author organized a meeting to resolve them through negotiation. The specific contents of the extracted data are as follows:

1. Article basic information: First author and year of publication.
2. Participant information: Participant characteristics including sample size, age, physical conditions (frailty and/or sarcopenia), and the diagnostic criteria used.
3. Intervention parameters: Specific implementation details of the experimental group’s interventions were extracted. Information on core intervention parameters (i.e., duration, frequency, intensity) was analyzed based on research requirements.
4. Outcome data: For the intervention outcomes of IL-6, CRP, and TNF-α, the pre- and post-intervention difference values between the experimental group and the control group were extracted. The data were presented as mean ± standard deviation (mean ± SD).Meanwhile, all three outcome measures are negative indicators.

### 2.4 Quality assessment

This study used the original Cochrane risk of bias tool (The Cochrane Collaboration’s tool for assessing risk of bias in randomised trials) (59), which was conducted independently by two reviewers (YSX and YND). Disagreements were resolved through discussion with the corresponding author. The following domains were evaluated:

1. Selection bias (random sequence generation): Assessment focused on whether true randomization was achieved in participant allocation, and whether the allocation sequence was effectively concealed prior to allocation completion. This concealment aimed to prevent researchers or participants from predicting group assignments, thereby avoiding selection bias.
2. Performance bias (deviations from intended interventions): Evaluation focused on whether the trial successfully implemented blinding for participants and intervention providers (e.g., physicians, nurses). If blinding was not achieved, the assessment determined whether systematic deviations in intervention delivery could impact outcomes.
3. Attrition bias (incomplete outcome data): Assessment determined the completeness of outcome data. If data were incomplete, it further evaluated whether the proportion and reasons for missing data were associated with true outcome values—this association would introduce bias into between-group effect estimates.
4. Detection bias (inappropriate outcome measurement): Assessment focused on the objectivity and reliability of outcome measurement methods, as well as whether outcome assessors were blinded to participants’ group assignments. Blinding was intended to prevent subjective judgments from being influenced by group information.
5. Reporting bias (selective reporting of results): Evaluation determined whether results reported in the final manuscript were consistent with pre-specified study protocols and analysis plans. It also checked for evidence of results being presented following “data mining” or “data cherry-picking.”
6. Other potential sources of bias: It logically integrates the assessment results of the previous five core domains rather than being an independent source of bias. The overall risk of bias is judged as “high risk” if at least one of the five core domains is rated “high risk”; with no domain rated “high risk”, it is “some concerns” if at least one domain is rated so; and it can only be “low risk” when all five core domains are rated “low risk”.

### 2.5 Statistical analysis

In this study, meta-analysis of the outcome measures of the included studies was conducted using RevMan 5.4 and Stata 15.1 software, to clarify the intervention effect of exercise on inflammatory biomarkers in older adults with frailty and/or sarcopenia. Given that all outcome measures of the included studies were continuous variables with inconsistent measurement units, statistical analysis was performed using the standardized mean difference (SMD) and its 95% confidence interval (CI).

In terms of heterogeneity assessment, the I² statistic and Q test were used for judgment: when I² ≤ 50% and p ≥ 0.01, it was judged as low heterogeneity; when I² > 50% and p < 0.01, it was judged as high heterogeneity. In cases of high heterogeneity, subgroup analysis and meta-regression analysis were employed to identify the sources of heterogeneity. Additionally, sensitivity analysis and publication bias detection were conducted to analyze the reliability of the research results. To evaluate potential publication bias, Egger’s test was used for statistical analysis in this study; if publication bias was detected, the trim-and-fill method was used to assess the stability of the overall effect of the study. In addition, sensitivity analysis was performed by excluding the included articles one by one to test the robustness of the meta-analysis results. Meanwhile, due to differences in the biological mechanisms of inflammatory biomarkers affected by different types of exercise interventions (such as aerobic training, resistance training, and comprehensive training, etc.), different intervention effects may occur. Therefore, subgroup analysis will be conducted to explore the differences in the impact of different exercise intervention types on outcome indicators.

## 3 Results

### 3.1 Studies search results

**Figure 1:**
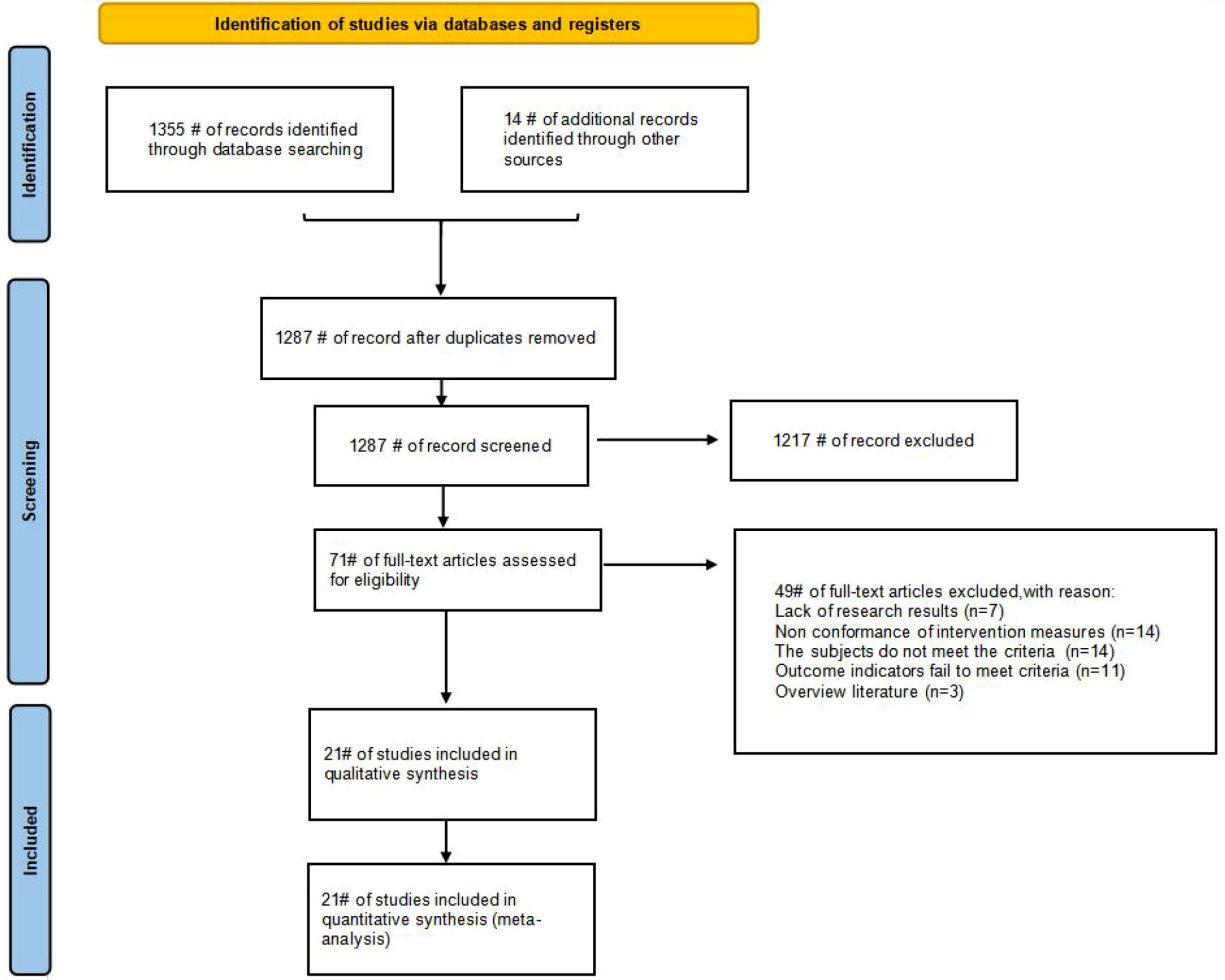
Studies selection process.

This study systematically searched multiple electronic databases and initially retrieved 1369 relevant records. First, duplicate removal was performed using EndNote reference management software, with 82 duplicate records excluded, resulting in 1287 remaining records. Next, preliminary screening was conducted by reviewing titles and abstracts; 1217 irrelevant records were excluded based on study topics and keywords. Finally, the remaining 71 records underwent full-text assessment in line with predefined inclusion and exclusion criteria, and 21 eligible studies were ultimately included. Notably, some of these 21 studies contained multiple groups with distinct exercise intervention modalities or frequencies, thus contributing 26 individual RCT (51, 52, 60–78). The study selection process is presented in **Figure 1**.

### 3.2 Characteristics of the studies

**Table 3** systematically summarizes the participant characteristics, grouping design, exercise intervention protocols, and relevant outcome measures of the 21 included studies (including 26 RCTs) (51, 52, 60–78). The total sample size across these studies was 1297 participants, including 659 in the experimental group and 613 in the control group. The mean age of all participants was approximately 72.8 years.

**Table 3:** Characteristics of included studies.

Study	Sample size (ETG/CG)	Age (ETG/CG)	Subject Characteristics	Intervention				Control group	Outcome
				Mode	Training movement	Intensity	Duration days/week (weeks)		
Zhang et al. (2025) (60)	ETG:60 CG:60	ETG:72.7 ± 4.8 CG:73.3 ± 4.6	Sarcopenia	AT	Eight postures were selected from the twelve-posture ancient Yijinjing: Veda Offering Pestle (two forms), Star-Plucking and Dipper-Swapping, Claw-Extending and Wing-Exposing, Reverse Pulling of the Nine Ox Tails, Nine Ghosts Pulling a Horse Saber, Three Disks Landing, and the Closing posture.	60% HRmax	5(12)	health education	IL-6;TNF-α;
Souza et al. (2022) (61)	ETG:14 CG:14	ETG:77.42 ± 6.25 CG:74.64 ± 7.13	Sarcopenia	RT	According to the American College of Sports Medicine (ACSM), the Resistance Exercise Training (RET) program for older adults should include eight exercises targeting major muscle groups: chest press, leg press, lat pulldown, abdominal crunch, leg extension, arm curl, leg curl, and arm extension.	50%-70% 1-RM	3(12)	placebo	IL-6;TNF-α;
Kim et al. (2016) (62)	ETG(1):36 ETG(2):35 CG:34	ETG(1):80.9 ± 4.2 ETG(2):81.4 ± 4.3 CG:81.1 ± 5.1	Sarcopenia	AT+RT	The experimental group adopted a combined training approach (aerobic + resistance). Aerobic training consisted of 12-minute stationary bicycle sessions (including 1-minute cool-down), starting at 40 watts with gradual progression, over three months. Resistance training involved progressive loading using hydraulic machines, including seated rowing, leg press, hip abduction, leg extension, and abdominal crunches.	–	2(12)	health education	IL-6;CRP
Silva et al. (2021) (63)	ETG:8 CG:7	ETG:80 ± 6.1 CG:86.7 ± 4	Frail elderly	AT+RT	RT: TheraBand resistance bands were used, with 8–10 exercises and progressive intensity/volume based on individual adaptation. Band color, length, and grip width were individually selected. AT: Aerobic and functional training included walking, stair climbing (up and down), and balance exercises.	RPE 4-6	2(32)	routine activities	TNF-α
Sadjapong et al. (2020) (64)	ETG:32 CG:32	ETG:76.68 ± 1.14 CG:78.87 ± 1.32	Frail elderly	AT+RT	The center-based intervention, guided by professional trainers, included strength, balance, flexibility, and endurance training (e.g., chair-based aerobic exercise), with safety guidance and exercise records.	40%-65% HRmax	3(24)	routine activities	IL-6;CRP
Heo et al. (2024) (65)	ETG(1):21 ETG(2):20 ETG(3):20 CG:20	ETG(1):67.81 ± 4.85 ETG(2):67.45 ± 4.70 ETG(3):67.95 ± 5.05 CG:67.30 ± 3.45	Sarcopenia	RT	The intervention included a progressive strength training program for older adults, with participants randomly assigned to low-, moderate-, or high-intensity groups (LSE, MSE, HSE). Training involved various equipment-based exercises (e.g., squats, bench presses, rows, leg presses), with weight progressively increased over months.	ETG(1):60% 1-RM ETG(2):70% 1-RM ETG(3):80% 1-RM	3(12)	Non-exercise	IL-6;TNF-α;
Huang et al. (2017) (66)	ETG:18 CG:17	ETG:68.89 ± 4.91 CG:69.53 ± 5.09	Sarcopenia	RT	Training covered all major muscle groups using Thera-Band^®^ resistance bands (color-coded resistance, 20% increase per level). Intensity was adjusted based on the Borg RPE scale: when participants reached an RPE of 13 (“somewhat hard”), they progressed to the next color; otherwise, they maintained the current intensity.	RPE 13	3(12)	routine activities	CRP
Chen et al. (2018) (52)	ETG:17 CG:16	ETG:66.7 ± 5.3 CG:68.3 ± 2.8	Sarcopenia	RT	The kettlebell training included 11 movements targeting major muscle groups (e.g., swing, deadlift, goblet squat, row, press, Turkish get-up, and dynamic training), progressing from basic to comprehensive movements, with chair assistance for injury prevention.	60%-70% 1-RM	2(8)	routine activities	TNF-α;IL-6;CRP
Nabuco et al. (2019) (67)	ETG:13 CG:13	ETG:68.0 ± 4.2 CG:70.1 ± 3.9	Sarcopenia	RT	The resistance training adopted a full-body protocol, including chest press, leg press, seated row, knee extension, preacher curl, leg curl, tricep pushdown, and seated calf raise.	–	3(12)	placebo	TNF-α;IL-6
Sang-Jung et al. (2024) (68)	ETG:14 CG:14	ETG:78.14 ± 3.72 CG:78.21 ± 3.72	Sarcopenia	AT+RT	The training included ten exercises (e.g., stationary walking, squats, lunges, jumping jacks, push-ups, crunches, hip bridges) plus warm-up and cool-down (10 min each). The main set consisted of 1 minute per exercise (10 min total), followed by a 5-min rest.	60%-85% HRR	3(12)	routine activities	TNF-α;IL-6;CRP
Tan et al. (2023) (69)	ETG:80 CG:57	ETG:73.39 ± 5.20 CG:71.69 ± 4.99	Frail elderly	AT+RT	It covers aerobic training, resistance training, dual-task training, and balance training.	–	3(12)	health education	IL-6;TNF-α;
Park et al. (2023) (70)	ETG:15 CG:15	ETG:79.7 ± 4.6 CG:81.5 ± 4.6	Frail elderly	AT	Course arrangement: It includes a 5-minute warm-up, 6 sets of stair exercise (5 minutes per set) with a 1-minute rest period between each set, and a final 5-minute cool-down.	50%-60%HRmax	3(8)	Non-exercise	CRP
Liu et al. (2025) (51)	ETG:30 CG:30	ETG:66.07 ± 4.05 CG:65.65 ± 4.6	Sarcopenia	RT	Each session included a 10-min warm-up, 50 min of resistance band training (elbow flexion/extension, shoulder abduction, bench press, seated row, hip extension/abduction, knee extension, standing calf raise, squats, performed in that order), and a 10-min cool-down.	SPE 6-7	3(12)	placebo	TNF-α
Alves et al. (2022) (71)	ETG:17 CG:15	ETG:70.6 ± 3.94 CG:71.4 ± 6.21	Sarcopenia	RT	It includes lower limb strength training exercises such as leg extension, 45° leg press, horizontal leg press, bilateral knee flexion (with weighted straps), hip abduction, and hip adduction.	50%-80% 1-RM	3(14)	placebo	IL-6;TNF-α;
Haider et al. (2017) (72)	ETG:35 CG:23	ETG:83.1 ± 7.8 CG:81.4 ± 8.7	Frail elderly	RT	Participants performed warm-up, then six exercises (mini squats, band chest press, seated abdominal training, standing hip extension, reverse butterfly, band shoulder press). Each exercise was done for two cycles of 15 repetitions until muscular fatigue.	SPE 6-7	2(12)	routine activities	TNF-α;IL-6;CRP
Ren et al. (2024) (73)	ETG:30 CG:39	ETG:70.4 ± 5.5 CG:71.9 ± 5.6	Sarcopenia	AT+RT	Aerobic exercise: 30 minutes of walking was used as the aerobic exercise modality. Resistance exercise: 40 minutes of resistance training was conducted using resistance bands.	–	3(12)	routine activities	IL-6;TNF-α;
Wang et al. (2019) (74)	ETG(1):20 ETG(2):20 ETG(3):20 CG:20	ETG(1):65.1 ± 2.8 ETG(2):64.2 ± 3.0 ETG(3):63.6 ± 5.2 CG:64.1 ± 2.8	Sarcopenia	ETG(1):RT ETG(2):AT ETG(3):AT+RT	RT: Progressive Resistance Training (ACSM-based) covering 8–10 major muscle groups (large-to-small muscle order), 10–15 reps × 3–5 sets. AT: Moderate-intensity aerobic exercise (AHA-based): 5-min warm-up stretching, 20-min dynamic aerobic rhythm exercises, 5-min cool-down. AT+RT: 10 min of RT followed by 20 min of AT.	RT:3–5 sets/10–15reps; AT:40%–60%,VO2max;	2(8)	Non-exercise	IL-6
Park et al. (2021) (75)	ETG:10 CG:9	ETG:66.6 ± 3.98 CG:64.8 ± 3.80	Sarcopenia	AT+RT	AT: Marching with shoulder abduction/adduction, alternating toe-touch walking, core stability/balance exercises, single-leg standing. RT: Supine pelvic tilt, partial abdominal crunch, alternating knee extension, kneeling bird-dog, plank hold.	50-60% HRR	5(15)	patients with osteoarthritis	TNF-α
Zhuang et al. (2025) (76)	ETG:13 CG:14	ETG:73.154 ± 4.22 CG:73.929 ± 3.73	Sarcopenia	RT	RT: 30 min session (5 min warm-up, 20 min RT, 5 min cool-down). Movements: shoulder external rotation, elbow extension/flexion, leg squat abduction, lunge and bend, shoulder abduction, half-squat stand-up.	60-70% 1-RM	3(12)	vibration training	TNF-α;IL-6;CRP
Ferreira et al. (2018) (77)	ETG:13 CG:14	ETG:73.3 ± 6.4 CG:77.8 ± 8.0	Frail elderly	AT+RT	The intervention included 3 weekly 40-min sessions covering muscle strength, speed, and agility. Training was personalized in small groups with professional guidance throughout.	SPE 5-7	3(12)	routine activities	IL-6;CRP
Lu et al. (2021) (78)	ETG:48 CG:150	ETG:69.95 ± 4.72 CG:69.98 ± 4.71	Sarcopenia	AT+RT	The specific exercise intervention included resistance training integrated with functional tasks, and balance training based on functional strength, sensory input, and attention demands.	SPE 5-7	2(24)	routine activities	TNF-α;CRP

ETG, Experimental group; CG, Control group; AT, Aerobic training; RT, Resistance training; HRmax, Heart Rate maximum; HRR, Heart Rate Reserve; VO_2max_, Maximal Oxygen Consumption; IL-6, Interleukin-6; TNF-α, Tumor Necrosis Factor-αlpha; CRP, C-Reactive Protein; 1-RM, One-Repetition Maximum; RPE, Rate of Perceived Exertion; SPE, Session RPE.

Among the included articles, 15 articles (20 RCTs) involved elderly subjects with sarcopenia (51, 52, 60–62, 65–68, 71, 73–76, 78),and 6 articles (6 RCTs) involved frail elderly subjects (63, 64, 69, 70, 72, 77). 10 articles (11 RCTs) adopted the exercise intervention method of combined aerobic training and resistance training (62–64, 68, 69, 73–75, 77, 78), 10 articles (12 RCTs) used resistance training as the exercise intervention method (51, 52, 61, 65–67, 71, 72, 74, 76), and 3 articles (3 RCTs) used aerobic training as the exercise intervention method (60, 70, 74). 15 articles (18 RCTs) included interleukin-6 (IL-6) as an outcome measure (52, 60–62, 64, 65, 67–69, 71–74, 76, 77), 10 articles (11 RCTs) included C-reactive protein (CRP) as an outcome measure (52, 62, 64, 66, 68, 70, 72, 76–78), 15 articles (18 RCTs) included tumor necrosis factor-α (TNF-α) as an outcome measure (51, 52, 60, 61, 63, 65, 67–69, 71–73, 75, 76, 78).

### 3.3 Risk of bias

**Figure 2:**
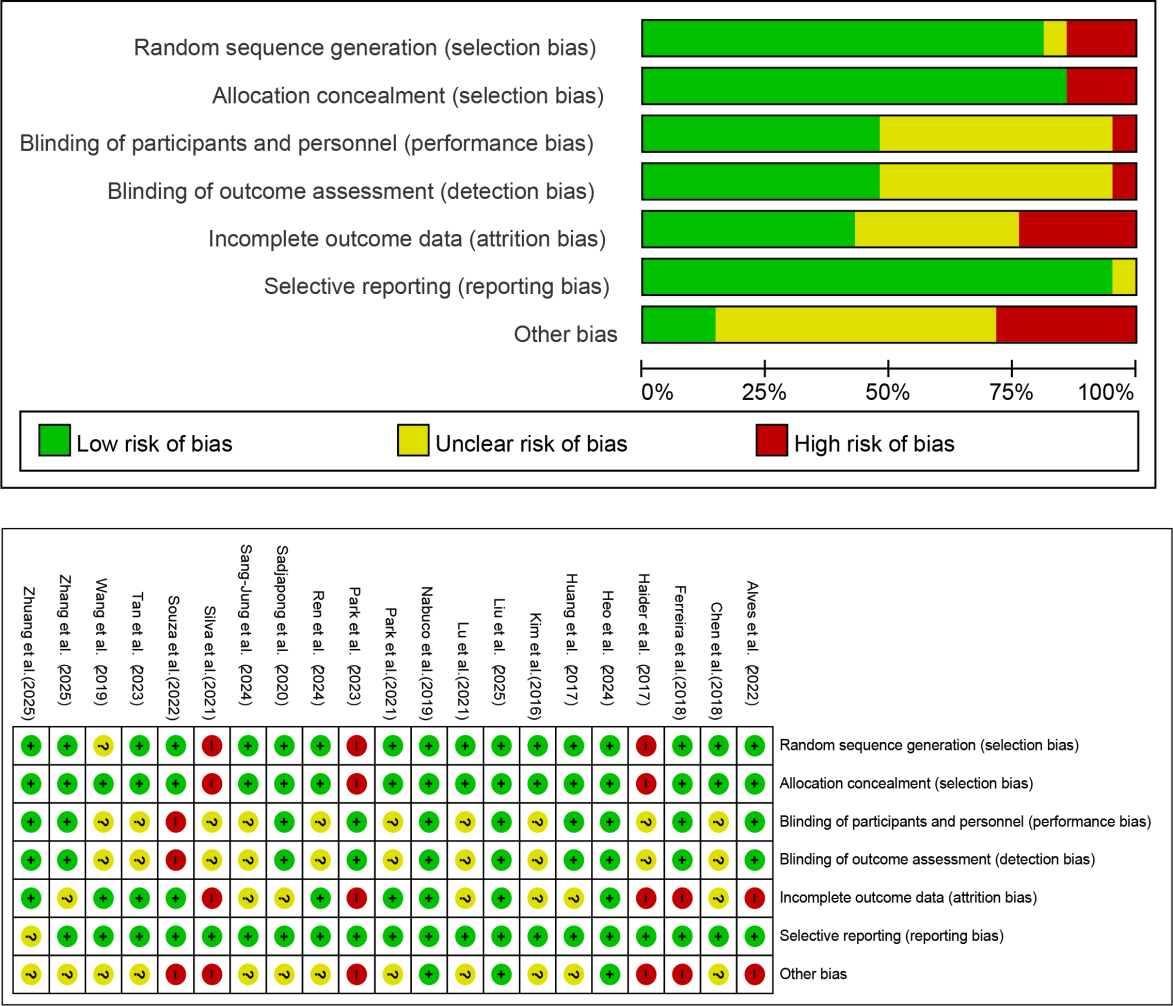
Risk of bias summary for included studies.

The risk of bias assessment results for the included studies are presented in **Figure 2**. Among them, 3 studies was rated as “low risk”, 6 studies were rated as “high risk of bias”, and 12 studies were rated as “unclear risk of bias”.

### 3.4 Meta-analysis results

**Figure 3:**
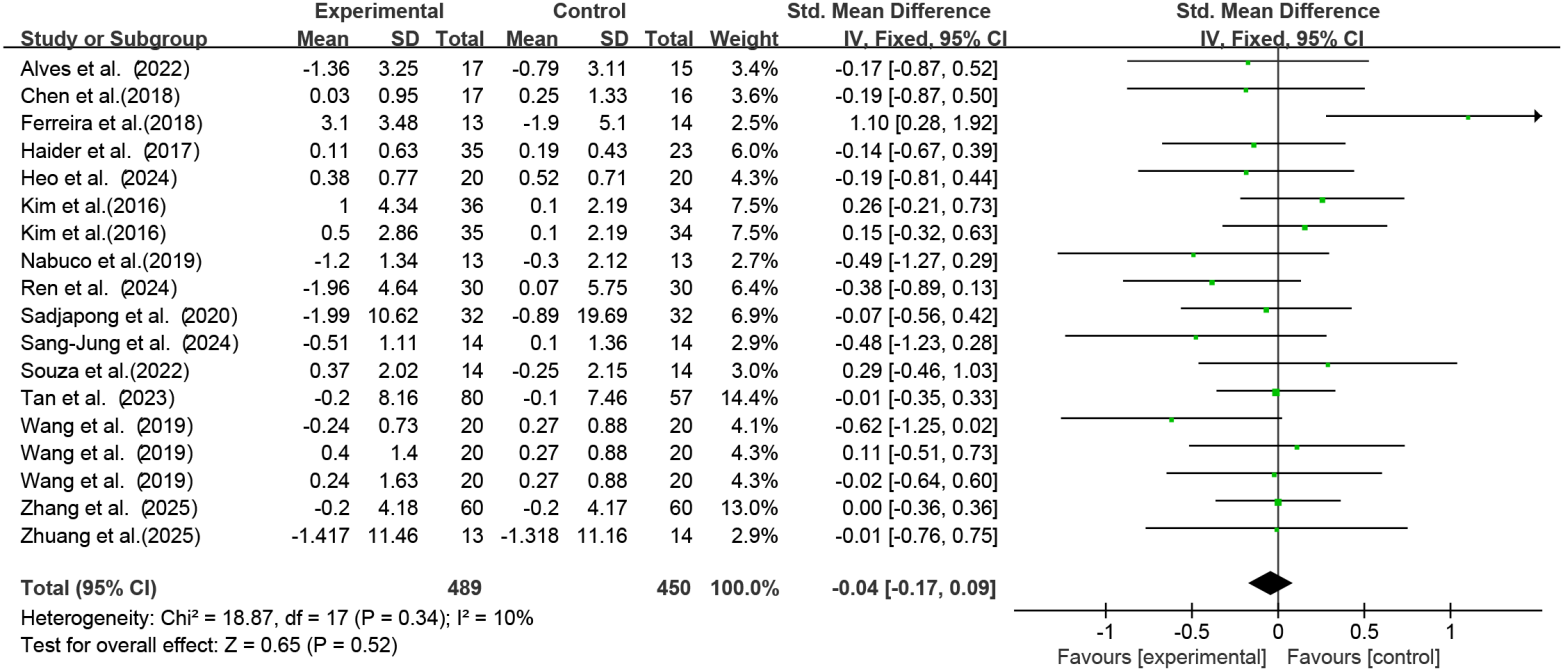
Analysis of the effect of exercise intervention on IL-6 in older adults with frailty or/and sarcopenia.

Among the included studies, 15 studies (18 RCTs) used exercise as the intervention to investigate its effect on serum IL-6 levels in older adults with frailty and/or sarcopenia. Meta-analysis (**Figure 3**) showed an effect size of SMD = -0.04 (95% CI: -0.17 to 0.09), *p* = 0.52; the difference was not statistically significant, indicating that exercise intervention had no significant effect on serum IL-6 concentrations in older adults with frailty and/or sarcopenia.

Heterogeneity assessment results revealed low heterogeneity across studies (I² = 10%, *p* = 0.34). Egger’s test was applied to assess publication bias among the 18 included studies, yielding a p-value of 0.795 (*p* > 0.05). This indicated no publication bias existed in the included studies, supporting the reliability of the results.

**Figure 4:**
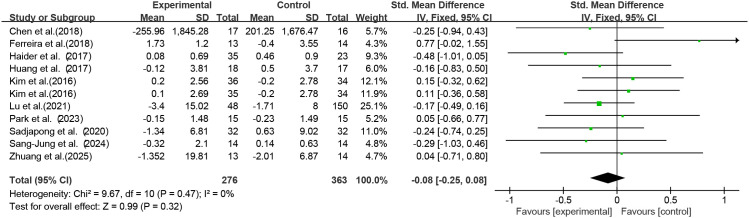
Analysis of the effect of exercise intervention on CRP in older adults with frailty or/and sarcopenia.

Among the included studies, 10 studies (11 RCTs) used exercise as the intervention to investigate its effect on serum CRP levels in older adults with frailty and/or sarcopenia. Meta-analysis (**Figure 4**) showed an effect size of SMD = -0.08 (95% CI: -0.25 to 0.08), *p* = 0.32; the difference was not statistically significant, indicating that exercise intervention had no significant effect on serum CRP concentrations in older adults with frailty and/or sarcopenia.

Heterogeneity assessment results revealed low heterogeneity across studies (I² = 0%, *p* = 0.47). Egger’s test was applied to assess publication bias among the 11 included studies, yielding a p-value of 0.470 (*p* > 0.05). This indicated no publication bias existed in the included studies, supporting the reliability of the results.

**Figure 5:**
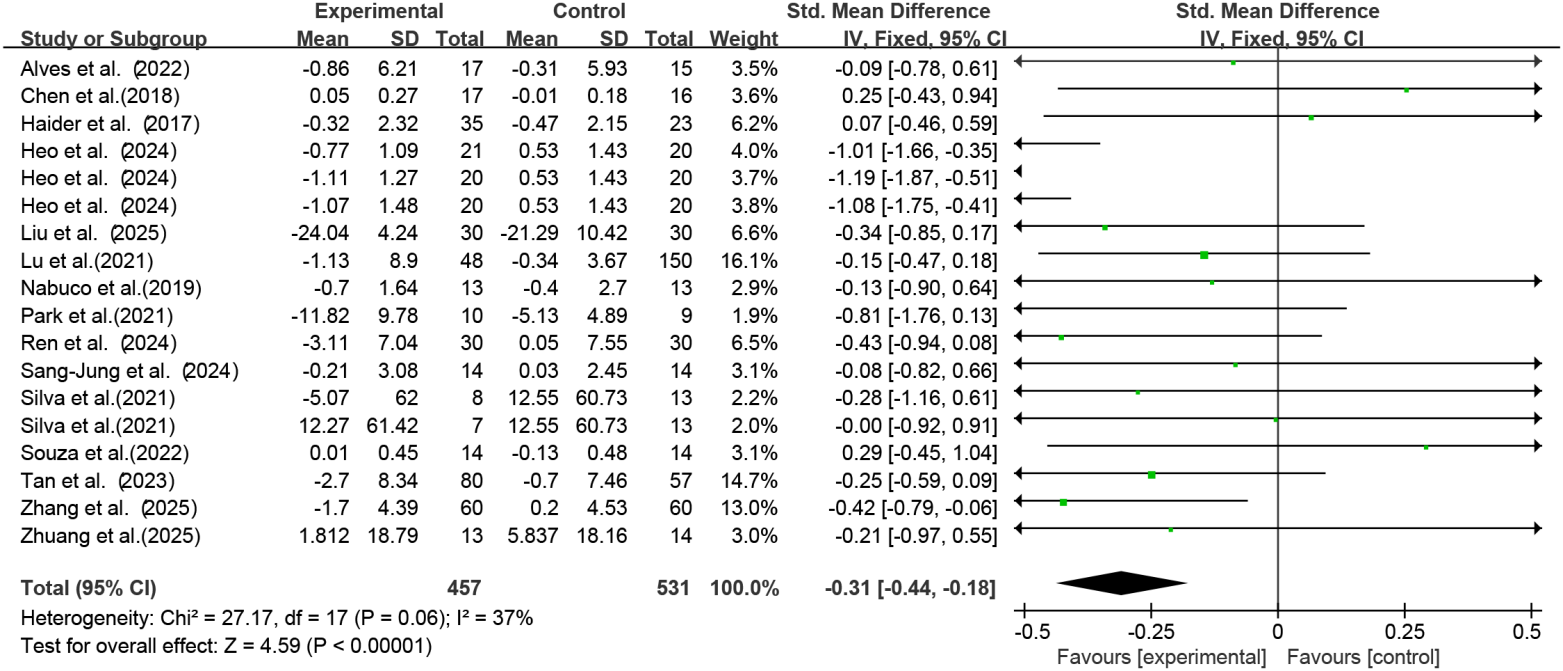
Analysis of the effect of exercise intervention on TNF-α in older adults with frailty or/and sarcopenia.

Among the included studies, 15 studies (18 RCTs) used exercise as the intervention to investigate its effect on serum TNF-α levels in older adults with frailty and/or sarcopenia. Through Meta-analysis (**Figure 5**), the effect size was obtained as follows: SMD = -0.31 (95% CI: -0.44 to -0.18), *p* < 0.0001. The difference was statistically significant, indicating that exercise intervention can significantly reduce the serum TNF-α concentration in older adults with frailty and/or sarcopenia.

The results of the heterogeneity test showed that there was moderate heterogeneity among the studies (I² =37%, *p* = 0.06). The Egger’s test was used to evaluate the publication bias of the 18 included studies. The results showed that *p* = 0.701, The results indicated the absence of publication bias, suggesting that the conclusions of this study have good reliability.

### 3.5 Subgroup analysis

Further subgroup analyses revealed (**Table 4**) that none of the three exercise modalities significantly improved serum IL-6 or CRP levels in older adults with frailty and/or sarcopenia. In contrast, all three exercise interventions demonstrated a significant beneficial effect on TNF-α levels, with AT showing the most pronounced improvement (SMD = -0.42, 95% CI: -0.79 to -0.06, *p* = 0.022). Notably,compared to those with frailty, subgroup analysis revealed that exercise intervention had a more significant effect on serum TNF-α in older adults with sarcopenia (SMD = -0.38, 95% CI: -0.60 to -0.16, *p* = 0.001).

**Table 4:** Subgroup analysis results.

Outcome	​Intervention parameters		n		Meta-analysis results		
			EG	CG	SMD	95%CI	*p*
IL-6	intervention types	AT	80	80	0.03	(-0.28,0.34)	0.863
		RT	149	135	-0.20	(-0.43,0.04)	0.099
		AT+RT	260	235	0.03	(-0.21,0.28)	0.790
	Subject Characteristics	Frailty	329	324	-0.08	(-0.23,0.08)	0.319
		Sarcopenia	160	126	0.11	(-0.28,0.51)	0.582
CRP	intervention types	AT	15	15	0.05	(-0.66,0.77)	0.886
		RT	83	70	-0.26	(-0.58,0.06)	0.113
		AT+RT	178	278	-0.01	(-0.24,0.23)	0.948
	Subject Characteristics	Frailty	95	84	-0.04	(-0.52,0.43)	0.855
		Sarcopenia	181	279	-0.07	(-0.26,0.13)	0.504
TNF-α	intervention types	AT	60	60	-0.42	(-0.79,-0.06)	0.022
		RT	200	185	-0.35	(-0.68,-0.01)	0.043
		AT+RT	197	286	-0.24	(-0.43,-0.05)	0.014
	Subject Characteristics	Frailty	130	106	-0.15	(-0.42,0.11)	0.247
		Sarcopenia	327	425	-0.38	(-0.60,-0.16)	0.001

To further explore potential sources of heterogeneity and to assess the robustness of the subgroup findings, meta-regression analyses were performed (**Table 5**). The results demonstrated that both exercise modality and participant condition were significant covariates associated with the effect estimates for TNF-α (*p* < 0.05 for both), thereby supporting the robustness of the primary subgroup analyses. For IL-6 and CRP, the meta-regression confirmed the absence of significant subgroup effects, consistent with the overall null findings. These results suggest that the observed improvements in TNF-α are reliably moderated by both the type of exercise and the underlying condition of the participants.

**Table 5:** Meta-regression results.

Outcome	Basis for subgroup analysis	β	SE	*t*	*p*	95%CI	
IL-6	Different exercise intervention types	2.338054	0.1683845	13.89	< 0.001	1.982793	2.693314
	Subject Characteristics	1.243553	0.1099973	11.31	< 0.001	1.011479	1.475627
CRP	Different exercise intervention types	2.48184	0.2043515	12.14	< 0.001	2.026517	2.937164
	Subject Characteristics	1.351269	0.1520067	8.89	< 0.001	1.012577	1.689961
TNF-α	Different exercise intervention types	2.315636	0.1504694	15.39	< 0.001	1.998173	2.633098
	Subject Characteristics	1.223051	0.1073443	11.39	< 0.001	0.9965744	1.449528

### 3.6 Sensitivity analysis

**Figure 6:**
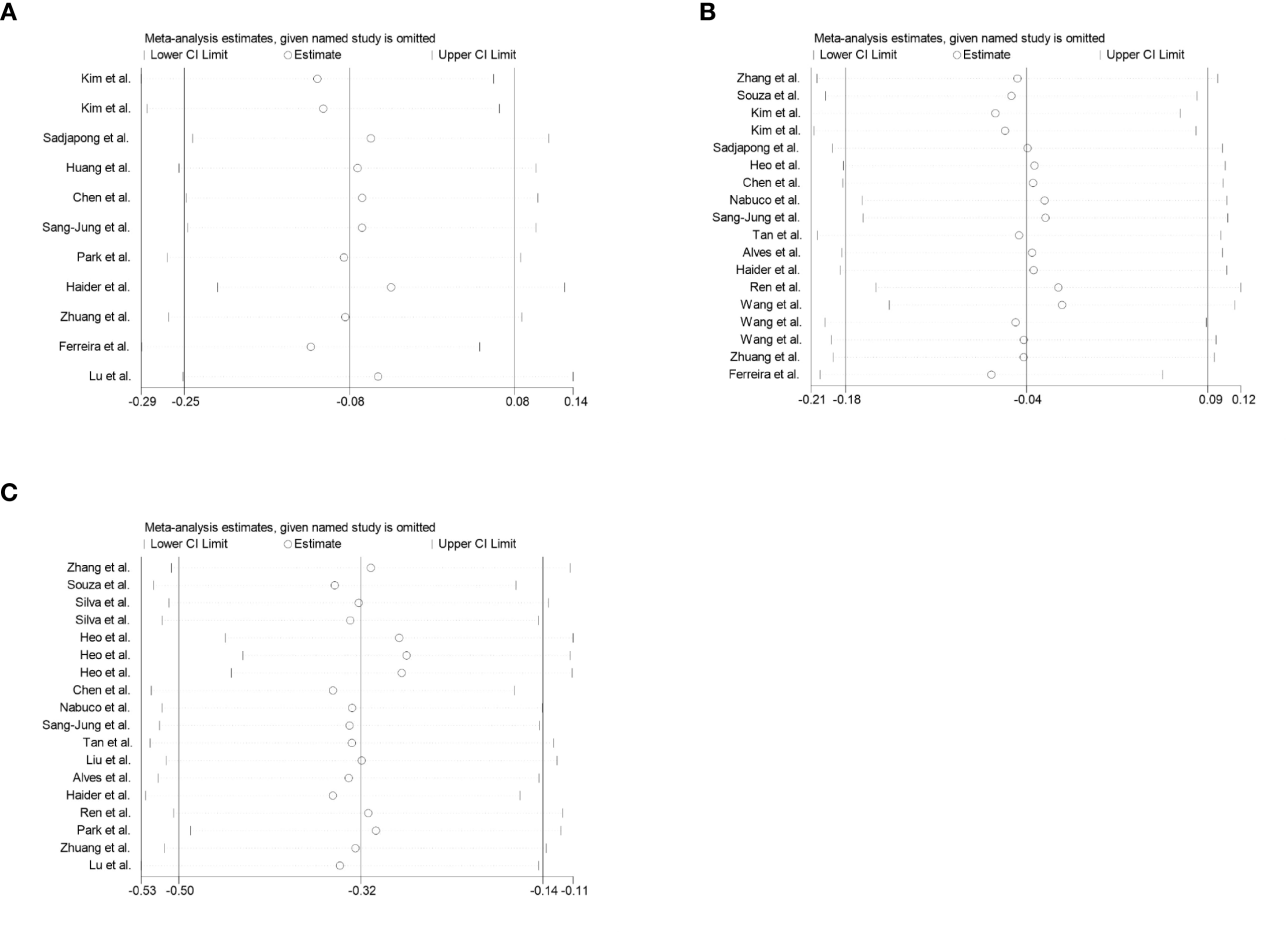
Sensitivity analysis plots for all outcomes. **(A–C)** correspond to: C-Reactive Protein(CRP), Interleukin-6(IL-6), Tumor Necrosis Factor-αlpha(TNF-α).

Sensitivity analysis was conducted by sequentially excluding each included study. The overall effect estimates remained stable, indicating that the meta-analysis results were robust and reliable (**Figure 6**).

## 4 Discussion

Using meta-analysis, this study synthesized data from multiple RCTs that targeted older adults with frailty and/or sarcopenia, with different exercise modalities as the intervention, to investigate the effect of exercise on serum inflammatory cytokines in this population. The results showed that exercise, as an intervention, did not significantly improve serum IL-6 and CRP levels in this group; however, it significantly reduced their TNF-α concentrations.

In previous research, meta-analyses focusing on the effects of exercise interventions on serum proinflammatory cytokines or inflammatory responses in older adults have been limited. For instance, a meta-analysis by Hoseinpour et al. focused on the effects of resistance training on inflammatory markers in healthy older adults. This study concluded that resistance training significantly improved serum CRP concentrations in healthy older adults (SMD = -0.74, 95%CI: -1.29 to -0.19, *p* = 0.008); however, it had no significant effects on TNF-α (SMD = 0.01, 95% CI: -0.45 to 0.47, *p* = 0.95) or IL-6 (SMD = -0.27, 95% CI: -0.61 to 0.07, *p* = 0.12) (79);Separately, a meta-analysis by Byrne et al. used serum inflammatory marker concentrations in older adults with sarcopenia or frailty as the primary outcome measure. Although this study concluded that the targeted interventions significantly improved IL-6 concentrations in this population (SMD = -0.28, 95% CI: -0.57 to -0.00, *p* = 0.05), the included interventions were highly heterogeneous—encompassing not only various exercise modalities but also multiple pharmacological or nutritional interventions. This heterogeneity prevented the isolation of the specific effects of exercise alone (80);Additionally, a meta-analysis by Sun et al. also focused on older adults with sarcopenia, but its primary objective was to investigate the effects of exercise interventions on body composition and muscle function in this group. Inflammatory markers were treated as secondary outcome measures, and the study included relatively few RCTs and a small total sample size. Furthermore, this study did not address heterogeneity across the included studies, which may limit its ability to accurately reflect the effects of exercise interventions on serum inflammatory cytokines in this patient population (81).

Compared with previous studies— which focused on healthy older adults, prioritized different outcome measures in their target populations, or included a range of interventions— this study primarily analyzes the effects of exercise training on serum inflammatory markers in older adults with sarcopenia and/or frailty. The results indicate that, for older adults with sarcopenia or frailty, exercise intervention did not significantly improve their IL-6 and CRP concentrations, but it exerted a significant intervention effect on TNF-α.Meanwhile, based on multiple characteristics such as exercise metabolic type, this study grouped different exercise interventions into AT, RT, and combined training (combining AT and RT). Through further subgroup analyses, this grouping aimed to clarify the intervention effects of different exercise modalities on different conditions (sarcopenia or frailty), thereby providing more intuitive evidence-based support and practical guidance for the development of public health policies, exercise prescriptions, and subsequent clinical trials.

## 5 Inflammatory biomarker and frailty, sarcopenia

With advancing age, damaged or stressed cells in the body (e.g., cells with telomere shortening or accumulated DNA damage) enter an irreversible growth arrest state known as cellular senescence (82). These senescent cells gradually acquire the Senescence-Associated Secretory Phenotype (SASP), which consists of a large number of inflammatory cytokines (particularly IL-6 and IL-8), chemokines, and proteases (83).

Numerous previous studies have shown that such persistently elevated inflammatory cytokines disrupt the dynamic balance between skeletal muscle protein synthesis and degradation through multiple molecular pathways, directly leading to muscle atrophy, which in turn contributes to the development and progression of sarcopenia or frailty. Specifically, the mechanisms by which inflammatory cytokines promote the development and progression of sarcopenia and frailty may involve the following: First, elevated levels of TNF-α and IL-6 are key signals that activate the Ubiquitin-Proteasome System (UPS) (84). By activating the nuclear factor kappa B (NF-κB) signaling pathway in cells, they significantly upregulate the gene expression of two muscle-specific E3 ubiquitin ligases: Muscle RING-finger protein-1 (MuRF1) and MAFbx (Atrogin-1) (85). These two ligases are responsible for recognizing and tagging sarcomeric proteins (e.g., myosin), directing them to proteasomal degradation—a process that represents the primary pathway of muscle catabolism;Second, inflammatory cytokines can antagonize key signaling pathways involved in muscle anabolism. They can inhibit the signaling of IGF-1 and induce insulin resistance (86). Notably, IGF-1 and its downstream Akt/mTOR signaling pathway serve as the “master regulator” of skeletal muscle protein synthesis. By inhibiting this pathway, IL-6 and TNF-α directly impair the ability of muscle cells to synthesize new proteins—thus hindering anabolism while simultaneously promoting muscle catabolism (87). Third, a chronic inflammatory microenvironment impairs mitochondrial function, which leads to reduced efficiency of adenosine triphosphate (ATP) production and increased generation of reactive oxygen species (ROS) (88). Excessive ROS directly causes oxidative damage to proteins, lipids, and DNA, further exacerbating muscle cell injury and apoptosis (89). Additionally, mitochondrial dysfunction and oxidative stress themselves can activate inflammatory pathways such as NF-κB, forming a local “inflammation-oxidative stress” vicious cycle (90, 91). Finally, the repair and regeneration of skeletal muscle depend on a population of muscle stem cells referred to as satellite cells. Studies have shown that a persistent inflammatory microenvironment—particularly in the presence of TNF-α—suppresses the ability of satellite cells to undergo activation, proliferation, and differentiation into mature muscle fibers, thereby severely impairing the muscle’s repair capacity following injury (92, 93).

Furthermore, the relationship between sarcopenia, frailty, and inflammation is bidirectional. Skeletal muscle atrophy and dysfunction are not merely consequences of inflammation; they also serve as a novel “inflammatory source” that conversely exacerbates systemic inflammaging, forming a self-reinforcing vicious cycle. Specifically, the potential mechanisms underlying this effect may include the following: First, in the muscle tissue of individuals with sarcopenia, not only muscle fibers themselves but also intramuscular stem cells (satellite cells) and fibroblasts undergo extensive senescence in response to stress (94). These senescent cells, which accumulate in the muscle microenvironment, sustainably secrete high levels of inflammatory cytokines such as IL-6 and TNF-α. This not only exacerbates local muscle catabolism but also allows their secretions to enter the systemic circulation, thereby increasing systemic inflammatory levels (95, 96). Second, the processes of muscle atrophy and fibrosis generate damage-associated molecular patterns (DAMPs), which recruit and activate immune cells (particularly macrophages) to infiltrate muscle tissue (97). These activated macrophages shift to a inflammatory phenotype (M1 subtype) and secrete large quantities of TNF-α and IL-6—transforming the muscle tissue itself into an active “inflammatory organ” and further facilitating the spread of inflammation from local to systemic levels (98, 99). Third, healthy skeletal muscle secretes a range of anti-inflammatory myokines (e.g.,IL-10, IL-1ra) during contraction (i.e., exercise). However, in the context of sarcopenia, reduced muscle mass and impaired physical activity result in decreased secretion of these beneficial anti-inflammatory cytokines (100). Finally, mitochondrial dysfunction and excessive ROS production are prevalent in the muscle of individuals with sarcopenia. As signaling molecules, ROS can diffuse extracellularly and activate inflammatory pathways (e.g., the NLRP3 inflammasome) in adjacent immune cells, prompting these cells to release potent inflammatory cytokines such as IL-1β. This process thereby converts signals of metabolic disturbance in muscle into systemic inflammatory signals (101).

## 6 Inflammatory biomarker and exercise

For the regulation of serum proinflammatory biomarker levels in the body, beyond pharmacological and nutritional interventions, a large body of empirical studies has shown that exercise training can effectively reduce serum proinflammatory cytokine levels in healthy populations or other populations without sarcopenia or frailty (102–105). The underlying mechanisms for this effect may include the following: Acute exercise—particularly prolonged, high-intensity muscle contractions—stimulates skeletal muscle cells to synthesize and release large amounts of IL-6, leading to a transient, sharp increase in its serum concentration (106). Notably, exercise-induced IL-6 can stimulate the release of other anti-inflammatory mediators, such as IL-10 and IL-1ra. As potent anti-inflammatory molecules, IL-10 and IL-1ra inhibit multiple proinflammatory signaling pathways, thereby forming a negative feedback regulatory loop that suppresses excessive inflammatory responses (107, 108). Meanwhile, studies have also shown that IL-6 can effectively inhibit the production of TNF-α by monocytes/macrophages stimulated by irritants such as lipopolysaccharide (LPS). As a key upstream initiator of the inflammatory cascade, TNF-α plays a pivotal role in triggering systemic inflammation; by inhibiting its production, exercise-derived IL-6 attenuates the systemic proinflammatory state at its source (109). In addition, as mentioned earlier, NF-κB is a core transcription factor that regulates inflammatory responses. When cells are exposed to proinflammatory stimuli (e.g., TNF-α), the IκB kinase (IKK) is activated—leading to the phosphorylation and ubiquitination of IκB, followed by its proteasomal degradation. The released NF-κB then translocates into the nucleus and initiates the transcription of a series of downstream inflammatory genes. In contrast, exercise can increase the activity of antioxidant enzymes and reduce the production of ROS, which is one of the key upstream signals for activating the NF-κB pathway (110).

Contrary to the findings of the aforementioned studies, this study synthesized a large number of RCTs investigating exercise interventions in older adults with sarcopenia or frailty by employing a meta-analysis approach. The results demonstrated that while exercise intervention could significantly improve serum TNF-α levels in this population, it did not exert a significant improving effect on CRP or IL-6. By synthesizing existing relevant studies, the potential reasons for this discrepancy in findings are considered to be as follows: A core pathophysiological feature of sarcopenia and frailty in older adults is inflammaging—an age-related, chronic, low-grade, systemic inflammatory state (111). The development of this state is closely associated with increased cellular senescence burden in the body. Senescent cells cease proliferation, acquire the SASP, and continuously secrete large quantities of inflammatory cytokines, growth factors, and proteases; among these, IL-6 is one of the most stable and core components of the SASP (83). In older adults with frailty and/or sarcopenia, a large number of senescent cells (present in multiple tissues including adipose, immune, and endothelial tissues) constitute a powerful, persistent endogenous source of inflammatory signals. While exercise interventions can elicit anti-inflammatory effects, these effects may be insufficient to counteract or “neutralize” the substantial and sustained surge of IL-6 (and its downstream mediator CRP) generated by SASP. In other words, the anti-inflammatory effects of exercise are of insufficient magnitude to counteract the persistent high-inflammatory state established by cellular senescence and thus fail to meaningfully modulate this pathological inflammatory background. Consequently, no significant reductions in serum IL-6 or CRP levels are observed.

Meanwhile, direct evidence explaining why TNF-α can be significantly improved by exercise in the present study remains lacking. However, the following speculations can be proposed based on existing theories: The primary chronic source of TNF-α in sarcopenic or frail older adults may be visceral adipose tissue (112). Exercise, particularly aerobic training, may reduce visceral fat content, thereby decreasing TNF-α production at its source (113). Additionally, exercise-induced myokine IL-6 may partially inhibit the secretion of TNF-α by monocytes/macrophages. These mechanisms may render the regulatory pathway of exercise on TNF-α relatively direct, resulting in a significant improvement effect at the meta-analytical level. Nevertheless, the above interpretations still require further validation in future research.

Furthermore, subgroup analysis in this study revealed that exercise training significantly reduces TNF-α levels in older adults with sarcopenia, but exerts no significant improving effect in older adults with frailty. This discrepancy may be attributed to the fact that inflammation in sarcopenia may be more closely associated with the local microenvironment of muscle tissue (10), whereas older adults with frailty not only present with muscle-related issues, but also frequently exhibit immune system dysfunction, neuroendocrine disruption, multi-organ dysfunction, as well as more severe systemic inflammation and a higher cellular senescence burden (114).Thus, in older adults with isolated sarcopenia, elevated TNF-α may primarily originate from dysfunctional muscle tissue itself or a small number of infiltrating immune cells (115). Exercise can act directly on muscle tissue to improve muscle metabolism, reduce local oxidative stress, and—via the myokine network (e.g., exercise-induced IL-6 inhibiting local TNF-α)—effectively decrease the production of muscle-derived TNF-α (116). In contrast, the sources of TNF-α in older adults with frailty are systemic and diverse. Beyond muscle tissue, a large proportion of TNF-α also derives from overactivated immune cells, dysfunctional adipose tissue, and the SASP of senescent cells distributed throughout the body (117, 118). When faced with such widespread and potent systemic sources of TNF-α, the direct interventional effects of exercise on muscle become attenuated, making it difficult to exert a significant impact on overall serum TNF-α levels.

In summary, existing studies have provided relatively clear insights into the mechanisms by which exercise improves inflammatory responses in the general population. These studies confirm that exercise exerts a significant anti-inflammatory effect in healthy individuals by regulating the dual roles of IL-6, inhibiting the NF-κB pathway, and other pathways. However, for the specific population of older adults with sarcopenia or frailty, the anti-inflammatory effects of exercise interventions are suboptimal. The underlying mechanisms for this limited efficacy remain unclear, and relevant research remains scarce—highlighting an urgent need for in-depth mechanistic exploration in this field. Based on existing theoretical deductions, this study posits that the limited response to exercise-based anti-inflammatory interventions in older adults with sarcopenia or frailty may be closely associated with “inflammaging” and the persistent high inflammatory burden induced by the SASP. Meanwhile, sarcopenia and frailty exhibit differences in the sources of inflammation and the extent of systemic involvement; this may further lead to distinct differences in the regulatory effects of exercise interventions on TNF-α between the two populations. Thus, this study holds dual value: On the one hand, by systematically evaluating the anti-inflammatory efficacy of exercise interventions in older adults with sarcopenia or frailty via meta-analysis, it provides a theoretical basis for the design of relevant clinical trials and the optimization of exercise prescriptions. On the other hand, by proposing innovative hypotheses based on existing theories, it offers research questions and practical directions for future studies.

## 7 Limitations

This study still has the following limitations:(a) Limitations in the quality of included primary studies Of the included primary studies, only 3 were rated “low risk”, with the remainder classified as “unclear risk of bias”. Several studies did not implement blinding or allocation concealment, which could introduce a certain degree of selection bias and performance bias, potentially affecting the reliability of their results.(b) Constraints related to sample size and population heterogeneity Although systematic searches were performed across major databases, a limited number of studies met the inclusion criteria. This study included only 1,297 participants (across 26 RCTs); notably, only 6 RCTs specifically targeted older adults with frailty, and only 3 RCTs employed aerobic exercise as the intervention modality. These small subgroup sample sizes may have reduced the statistical power of some findings. Furthermore, sarcopenia-related comorbidities (e.g., sarcopenic osteoporosis, sarcopenic obesity) were not further stratified, potentially resulting in inadequate consideration of disease-specific clinical manifestations and their potential impact on outcomes.(c)Insufficient standardization of exercise intervention parameters. The included studies showed significant heterogeneity in exercise intervention parameters, including duration (i.e., intervention cycle), session length, intensity, and frequency. This high variability hindered the extraction of an optimal dose-response relationship between exercise and improvements in proinflammatory cytokines, limiting the ability to define evidence-based exercise protocols.(d)Inadequate assessment of long-term intervention effects. The included studies had no long-term follow-up data available, preventing the effective assessment of the sustainability and long-term effects of exercise interventions. It remains unclear whether the observed improvements in inflammatory biomarkers can be maintained over time, which limits the clinical applicability of the findings for long-term geriatric care.

To address the limitations identified in this study, future research can be advanced and refined through the following strategies:(a) Enhance study design rigor. Priority should be given to conducting multicenter, large-sample randomized controlled trials (RCTs) with standardized exercise intervention parameters. Specifically, parameters such as exercise intensity (e.g., quantified via percentage of maximum oxygen uptake or Borg scale), single-session duration, weekly frequency, and total intervention cycle should be clearly defined and harmonized across studies to reduce inter-study heterogeneity. Additionally, rigorous protocols for blinding (where feasible) and allocation concealment must be enforced—including training for researchers on implementation procedures—to ensure methodological transparency and minimize selection or performance bias, thereby strengthening the reliability of findings.(b)Extend trial duration and follow-up. Exercise intervention trials should be designed with a minimum duration of 48 weeks, coupled with long-term follow-up assessments (e.g., 6 months and 1 year post-intervention). This will enable evaluation of not only the sustained effects of exercise on inflammatory biomarkers but also its role in delaying the progression of sarcopenia and frailty. Such data are critical for establishing evidence-based guidelines for long-term exercise adherence in geriatric care.(c) Explore combined intervention strategies. Future studies should investigate synergistic effects of exercise combined with nutritional or pharmacotherapeutic interventions. For nutrition, research could focus on clarifying how specific supplements (e.g., high-quality protein, omega-3 fatty acids, vitamin D) modulate the anti-inflammatory efficacy of exercise. In pharmacology, attention should be paid to whether exercise can mitigate medication-related adverse effects (e.g., muscle wasting associated with glucocorticoids or certain anti-cancer drugs) while enhancing therapeutic outcomes.(d)Deepen mechanistic investigations. Building on the current findings, further research is needed to unravel the underlying physiological and biochemical mechanisms by which exercise regulates serum inflammatory cytokines in frail or sarcopenic older adults. This could include exploring key pathways such as the NF-κB signaling cascade, MAPK pathway, or the role of myokines (e.g., irisin, BDNF) released during exercise. Elucidating these mechanisms will inform the design of targeted exercise prescriptions and personalized intervention strategies, maximizing the anti-inflammatory and functional benefits of exercise for this population.

## 5 Conclusion

This study employed a meta-analysis approach, where the results of existing RCTs were systematically synthesized and quantified. The findings revealed that while exercise intervention failed to significantly reduce serum IL-6 and CRP concentrations in older adults with sarcopenia and/or frailty, it was found to significantly reduce their serum TNF-α concentrations. Meanwhile, further confirmation via subgroup analysis showed that three exercise modalities—RT, AT, and combined training (AT + RT)—all effectively improved serum TNF-α concentrations in this population, with aerobic training demonstrating the optimal effect.

The exercise protocols that yielded significant improvements in TNF-α among the included studies typically fell within the following ranges. These parameters are summarized from the effective interventions observed in this meta-analysis and may offer preliminary reference points for clinical application:

AT: 40%–65% HRmax or HRR, 3–5 sessions/week, 30–60 min/session (e.g., brisk walking, cycling); RT: 50%–80% 1-RM or RPE 4–7, 2–3 sessions/week, 8–10 multi-joint exercises (e.g., leg press, chest press), 2–4 sets of 10–15 repetitions; AT + RT: 2–3 sessions/week, 40–60 min total (10–30 min RT + 20–30 min AT).

These ranges represent a synthesis of effective intervention parameters from the included trials. However, as this meta-analysis did not conduct dose-response modeling, these values should be viewed as observational summaries rather than definitive prescriptions. They provide a practical foundation for future research and for designing exercise interventions in community and geriatric settings, particularly for targeting TNF-α reduction in this population.

## Data Availability

The original contributions presented in the study are included in the article/Supplementary Material. Further inquiries can be directed to the corresponding author.
